# Gamo's cultural forum-Dubussha as a tool for social change communication: Illiteracy and child labor abuse as a case in point

**DOI:** 10.1016/j.heliyon.2024.e27674

**Published:** 2024-03-11

**Authors:** Mekonen Teka Ayalew, Tafesse Walea Wanke

**Affiliations:** a Media and Communication at Arba Minch University, Department of Ethiopian Languages and Literature-Amharic, Ethiopia; b Computational Linguistics at Arba Minch University, Department of Ethiopian Languages and Literature-Amharic, Ethiopia

**Keywords:** Grass-root communication, Dubussha, Illiteracy, Child labour abuse, Social change communication, Alternative knowledge, Participatory approach

## Abstract

This study investigated the instrumental potential of Gamo's traditional dialogue forum-Dubussha-for social change communication in general, using illiteracy and child labour abuse as a case in point. The study went on to analyze the motive that when traditions pave the way for new ways of life and thinking, changes are more applicable and transformative. As a result, this study sought to apply current values in a more local context and discovered that using culture as a vehicle for transformation yielded positive results. The two most popular tactics used to satisfy the study's goal of gathering information from largely alternatively educated respondents were intensive interviews and focus group discussions. Since cultural societies' skills, expertise, and knowledge are intricately related to their culture, the qualitative technique was effective in understanding and testing the research questions of this study. According to the study's objectives, three different focus group discussions (FGDs) were held in the Dita, Daramalo, and Chencha districts. Participants in the focus groups ranged in age, gender, and cultural background. Each FGD session included a diverse variety of participants, including women, men, student participants, and cultural leaders (Haleqas and/or Hudugas (opinion leaders). The FGD had 27 participants until it was full. Participants, facilitators, and in-depth interviewees were also purposively chosen. Finally, the study's findings indicated that Dubussha had a large potential for social change communication, particularly to reduce illiteracy and child labour abuse. Given that the cultural communication forum, Dubussha, is the major pillar of Gamo society's psychosocial structure, its use as an effective instrument for social change communication produced significant outcomes. Despite the gender and age differences in the application of "Dubussha," the outcome, acceptance, and glory of Dubussha among the Gamo community remained consistent.

## 1 Introduction

The importance of paying close attention to development issues is not a new concept. It was widely accepted in the 1950s and early 1960s, when global development was becoming a major topic and the focus of academic research. However, keeping a record of the concepts that traditionalism was an issue of underdevelopment was a key element of the discussion [1,2]. The solution appeared to be as obvious: replicating and cultivating modern technology, modes of production, institutions, and, eventually, habits and values from the West to the Non-West via mass media [[2], [3], [4], [5]]. It was expected that wealthy countries and magic multiplier media would regulate development [6,7]. However, this brief and flimsy definition of development did not contribute to closing the gap between rich and poor states, or even between affluent and poor people within third-world countries.

Accordingly, South countries fell deeper into poverty, compelling influential diffusion model experts [8,9] to reconsider their models' foundations in order to incorporate the cultural issues of third-world countries [8]. Later, development was envisioned as a broadly participatory social transformation process in which the majority of a society has control over its surroundings. It is a dynamic force that mobilises and enables rural, diverse, variously educated, and tradition-bound groups in emerging countries to obtain access to the pathways of already developed nations.

Although approaches to developmental path ways have been thoroughly studied, the complexity and breadth of the presentation were questioned until current empirical research and the consulted literatures. Unfortunately, the have-nots were either oblivious of or unable to contribute to global development through their customs and world views. It is apparent that social transformation can be accelerated by grassroots participation and systematic use of alternative media. As a result of their alternate change platforms, they can better their lives rather than waiting for progress somewhere else. Thus, communicating social change using different ways to communicate like Dubussha is critical for long-term intervention in a place with a more culturally dominant population like Gamo in Ethiopia.

Communication has been and continues to be important for social change; both the panel and those on the floor should contribute equally for a variety of reasons, particularly in developing countries where reliance on mainstream media may not be sufficient to achieve true development. Its impact and influence may be limited since it is not easily available and in a position to deliver credible and relevant information to the grassroots. Although it is considered that the mainstream media is filled with modern culture and civilization, it appears strange to individuals living in undeveloped countries and slums. The process is also heavy handed by urban dwellers, while those who need development the most; the poorest of the poor, slum dwellers and marginalized groups are eluded. Intensely, one of the major discoveries for US researchers in 'third world' countries is that local people have their own knowledge base and these can be powerful change agents if properly utilized [10,11]. When dealing with the remains of modernization, more recent generations of theorists and practitioners are tempted to seek grounding in tradition [12]. Folk media are the creative dissemination of information through the arts of culture and performance. In traditional societies, folk media: drama, skits, poems, stories, riddles, songs, and dance have been popularly and successfully used to disseminate messages and even to pass on the wisdom of older generations to the youth [13].

In in compliance with the principles of grassroots inclusiveness and participatory communication approaches, cultural communication forums such as Dubussha of the Gamo people of Ethiopia are believed to have provided an urgent solution to the local problem of child labour abuse and illiteracy reduction intervention in Gamo zone, Ethiopia. Furthermore, social problems in Ethiopia in general and the Gamo Zone in particular, are firmly founded and ingrained in cultural connection. As a result, using media (print, radio, telephony, video, and the Internet), education (training, literacy, and schooling), or alternative communication forums like Dubussha to intervene in a systematic or strategic manner to effect positive societal change is unquestionably critical. The intervention modality can be economic, personal, cultural, sociological, or political [1], and including a cultural element as a change agent has undeniably substantial results. Thus, in accordance with the above theoretical assumptions, this research will address the following primary questions.

➢ Why is using the Gamo Dubussha for communication of social change important?
➢ What features of Gamo's Dubussha have an effective potential for communicating social change?
➢ How can cultural forums be a solution to social problems such as illiteracy and child labour abuse?

### 1.1 A brief socio-demographic account of the Gamo people

The Gamo people of Ethiopia are known for their peace loving culture and tolerance. However, in child labour abuse and working on the reduction of illiteracy there is a gap that needs intervention through the use of their cultural discussion forum Dubussha in a more sustainable and long-lasting way. There are two meanings of the word "Gamo" according to the elders of the ethnic group. Gamo are strong, wise, honest, and respectful, according to their first myth. However, the second meaning is "lion". Gamo has inhabited southern Ethiopia for a long time. They are one of Ethiopia's most culturally diverse ethnic groups. Their culture and nature merge seamlessly here. The Gamo language is spoken by 1,044,589 people [14] who live in the south-west of Ethiopia. It is classified as Afro-asiatic, North Omotic, and Gonga-Benoyem in the genetic classification of Ethiopian languages [15].

The traditional government system (Dere system) has played a big role in the Gamo people's long political history in the age-old formation of a profound cultural make-up. Furthermore, the discussion platform (the Dubussha procedure) has been particularly helpful in Gamo decision administration and interventions. Gamo's Dubussha, one of the most respected and colourful social traditions, provides people with both pleasure and a welcoming environment in which to discuss local and/or regional "Dere"-related minor and major issues. Solving conflicts through the gathering at Dubussha is a fundamental and necessary element of Gamo life, with significant implications for peace and unity.

In a broader sense, knowing the Dubussha components sympathetically means knowing the Gamo people well. Any intervention that does not take into account this longstanding custom is unlikely to succeed. For a long time, the Gamo people have valued and respected Dubussha as a location, a gathering place, and a site of practice. This communication channel could be used to debate any subject, social, cultural, or even personal. People in Gamo's culture who breach the Woga system's prohibitions known as "Gome" are cursed and face harsh punishment in "Dubussha" sessions that are said to be passed down from generation to generation. The traditional laws and regulations were perceived to have begun with the creation of Earth, which they refer to as the "Wonta Woga/Divine law." These cultural foundations and theoretical understandings helped to solidify the idea that employing traditions in more inventive and systemic ways has a beneficial social change impact.

## 2 Significance of the study

Several interventions in the study area have been identified as failing as a result of the approach used by researchers. As it does not take cultural factors into consideration, the imported strategy has not been as successful as intended. However, according to this study, the failure of previous efforts was due to a lack of integration of those local wisdoms. This feature is closely related to the major benefit of the research findings discussed in this study. As proven in this study, combining cultural settings with contemporary ideas and approaches produces significant effects. More specifically, this study intends to fill a knowledge gap that is thought to be vital for future development communication interventions in more culturally dominant nations.

## 3 Limitation of the study

The study has the following main limitations, despite its strengths in highlighting and introducing why, what, and how to handle developmental problems in the culturally dominated context of developing countries.

➢ Its scope and concept lack depth.
➢ It involved a limited sample & population
➢ It uses small time-frame interventions due to limitation of finance & logistics.

## 4 Methods of the study

The research is entirely qualitative in nature. According to Refs. [16,17], qualitative research is useful in researching a social phenomenon, exposing emotions associated to the problem, and comprehending the individual experiences of persons involved in the study problem. The technique involves in determining how people interpret their social situations. The methodology has acquired favour in social science studies because to its ability to describe social reality [18]. As a result, utilising the cultural communication forum Dubussha as an instrument, a FGD and an interview were utilized as part of qualitative research methods to collect data and comprehend societal problems, including illiteracy and child labour abuse in Gamo people in Ethiopia. The researchers attempted to identify whether their cultural values are instrumental in their social problems through repeated interviews and focus group discussions. Furthermore, triangulation was accomplished using observation, reducing the approach constraint.

In fact, prior to conducting human subject research the research was approved by the institute's ethics committee. The experiments were then carried out in accordance with established ethical guidelines, and informed consent was obtained from participants under the supervision of the research's sponsoring institution, Arba Minch University's College of Social Sciences and Humanities, Ethiopian languages, and literature-Amharic research Ethics Committee. Therefore, all data used for research writing purposes, as well as files for this evaluated and approved research, can be found in the file named AMU-TH-22-CSSH-DELLA-01-2012, particularly, the ethical clearance reference number is DELLA-155-15. Furthermore, in response to requests, it is likely to provide details of the ethics clearance committee that approved these experiments, along with confirmation that the study complies with all regulations and that informed consent was obtained. Individuals' written consent to have their photographs used in this research was also confirmed, in accordance with the university's research ethics guidelines.

### 4.1 The FGD

FGDs are only as effective as the moderator, who needs to be able to think, listen, and manage time simultaneously [19]. In this role, he/she must ensure that the discussion topic is clearly introduced, is discussed, and that the discussion is balanced and inclusive. Despite this crucial role, the moderator should avoid dominating the group and expressing his/her own judgments. As a result, he/she should be open, alert and probing, and encourage everyone to participate in the discussion. Ideally, the moderator should be able to establish a group dynamic in which participants discuss topics from the discussion guide among themselves, rather than relying on the moderator to address and interview participants, one by one [20].

In order to achieve the purpose and objective of this research, three different FGD sessions have been held in the Dita, Daramalo, and Chencha areas. According to the objective of the study, the participants of the FGD were drawn from various age groups, sexes, and cultural backgrounds. For each FGD session, two women, two men, two student participants, and five cultural leaders (Haleqas/Hudugas (opinion leaders)) participated. Up until the saturation stage, 27 participants participated in the FGD.

As in any qualitative research, here are a few steps that this research has taken, which are usually taken when designing and implementing FGDs [21]. Select the team that will facilitate the discussions, STEP 2: Determine which groups and participants will participate, STEP 3: Determine the timing and place of the meetings, STEP 4: Develop a set of questions or a discussion guide, STEP 5: Conduct and record the focus groups, STEP 6: Analyze the data.

### 4.2 An interview and procedures of the intervention

In this study, the interview is the second method. More importantly methods such as this one are employed because it is the most effective way to collect data from largely unschooled individuals. When a researcher needs to gain insight into people's opinions, feelings, emotions, and experiences, interviews are almost certainly the preferred method [22]. The interview is advantageous because the language of the interview can be adapted to the ability or educational level of the person interviewed, and as a result, misinterpretations about the questions can be avoided [23]. Events that have significant social implications often occur so rapidly or unexpectedly that researchers cannot observe behaviour at the time. In such cases, an interview is an effective weapon [24]. Thus, the interview is an effective method of studying trends, potential, and the use of traditional communication tools in the Gamo Zone for development.

One of the two researchers for this study grew up and lived in the Gamo Zone Chencha area. Furthermore, since both researchers work in this community, they have little trouble acquiring data. Purposive sampling was used to choose trainee trainers who specialised in sociology, anthropology, and development communication for the intervention portion of the project. In this study, three trainee trainers were responsible for training five FGD opinion leaders prior to an open FGD session in each study location. Covert training was used to choose those five opinion leaders who are thought to be opinion leaders using snowball sampling. The remaining twenty-two FGD participants were purposefully included and interviewed after and before the FGD in order to track the changes observed as a result of the intervention. In-depth interviews were also conducted using purposeful sampling. Hudugas, Haleqas, Native Gamos, Culture and Communication Experts, Non-Governmental Organizations (NGOs), and Development Experts were also interviewed, and their perspectives were recorded for thematic analysis.

### 4.3 Method of data analysis

It is clear that the choice of the data analysis approach is mainly determined by the study tools. This study, which is qualitative in nature, used thematic analysis as a qualitative method of analysis. Thematic analysis is actually an ideal technique for studying data, much like research that examines information gathered from focus groups, in-depth interviews, and observation. This study used the thematic data analysis method, which is becoming more and more common for analysing qualitative data. The themes were produced using the qualitative instruments discussed above and the themes were coded and categorised.

## 5 Data presentation and discussion of findings

Based on the research questions and the data collected from the field, three main themes have been emerged. In the data presentation and discussion part of the study, grand themes and subthemes were also systematically incorporated and analyzed. The first theme addresses "Dubussha" in terms of the cultural or social constructs relevant to it in the Gamo community. A second theme focused on the many attributes of "Dubussha" that are meant to elucidate the current trend of its use. In the last and the main part, it discussed the communicative potential of "Dubussha" though reducing illiteracy and child labour abuse in Gamo Zone as a case in point. Furthermore, the themes are presented in a manner in which the preceding theme develops the next (Table 1).

**Table-1:** aRespondents' identification code.

S.N	Code	Description
1	HHG	Halaqa and Huduga of Gamo People
2	OGE	Ordinary Gamo Elder
3	OGF	Ordinary Gamo Female
4	CCE	Culture and Communication Expert
5	CLE	Child Labor Expert
6	CEE	Child Education Expert
7	FN	Field Note

a Table 1 displays the codes used in the analysis of qualitative data from this investigation, which was assembled and coded by the researchers.

### 5.1 Definition of “Dubussha”: natives' point of view

Dubussha and what it means for the Gamos have been defined by the HHG in the following way:

Since the creation of the Earth, the both 'Wonta Woga' and the Dubussha has served as a place of ritual practice, a value, a rule, and a traditional government system. In Dubussha, for example, you will find a green area surrounded by mountains and the tombs of the ancestors of that community. In the Gamo community, no problem is beyond the control of the Dubussha and no one is beyond the decision of this assembly (*FN1, p. 1).

#### 5.1.1 Essence of Dubussha: looking at it through the eyes of people

Compared to the modern channel of influence, "Dubussha" may seem an inappropriate practice for people who are not Gamo. It looks like the old way of gathering people for modern problems in cities. Today, however, the Gamo People who are living in civilized nations give the same definition like the traditional Gamos. They emphasize the importance of their cultural values to communicate a number of issues. They also strongly argue that whatever their educational level is, their personality is highly influenced by their culture (CCE, FN 3).

As (CCE, FN 4) continued in its description, few non-Gamo people living in the study area see the practice of "Dubussha" as a manifestation of traditionalism and an uncivilized form of communication. Others, however, viewed it as an alternative way to transform the current culture-oriented society into a modern social and political order. The Gamo people are now considered as a symbol of friendship and fraternity in Ethiopia, where ethnic tensions persist. Across the country, there are many people who can give their consent about the peacefulness of the Gamo people that is because of the positive influence of the Dubussha (Fig. 2).

**Figure 1:**
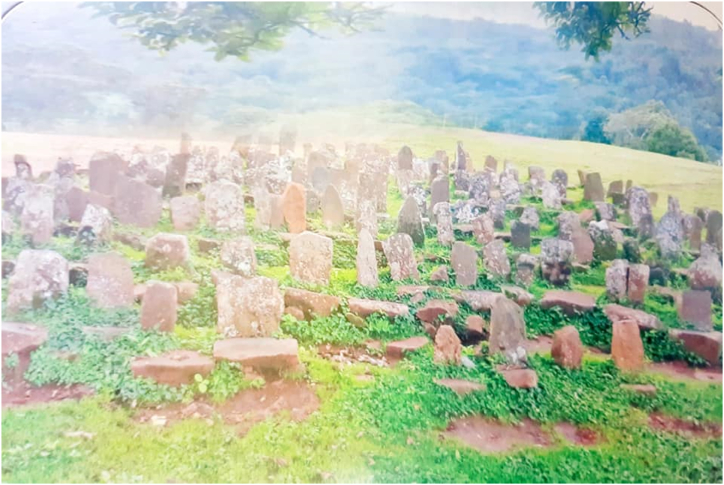
On-location photo taken by researchers^1^.

**Figure 2:**
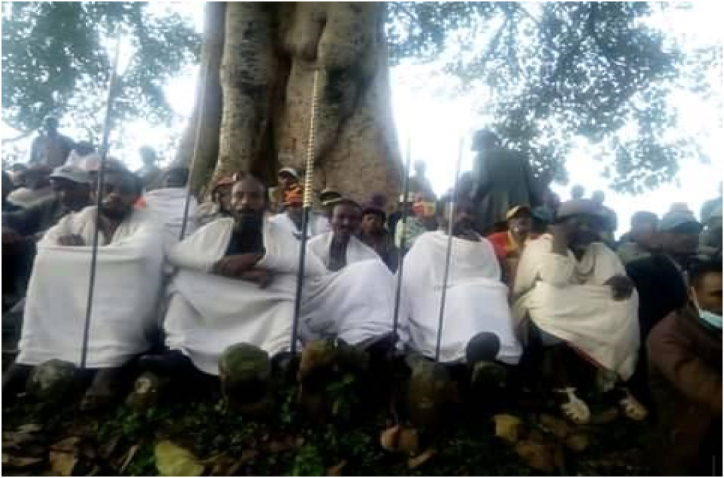
(taken by the researchers with the consent of the local community)^2^.

HHG has also explained why the Gamo's value and norm are so important, even at the national level:

Gamo as a person is an embodiment of co-existence, a person who is truly human, selfless, and humble in all facets of his life. Kindness and humanity are highly associated with culture and the value of society at large. Dubussha is one of the most precious cultural treasures of a person. Personally, I believe that Gomo culture should be expanded and adapted throughout the country. (FN1 p. 5) Haleqas and Hudugas of the Gamo people claimed that their statement has a great impact and unwavering acceptance in the Gamo community due to the “Woga Wonta”.

Additionally, the rules and restrictions in "Woga Wonta/ Divine power" are the very essence of the Gamo way of life. Since the birth of the planet, we have been dedicating ourselves to hard work, dialogue, cooperation with nature, including humans, and governing by the law of the land. Regardless of age or educational level, the values of society are crucial to living in harmony and dignity with any group of people (HHG, FN1, p. 6)

#### 5.1.2 It is the essence of self-expression and confidence

Through Dubussha and its impacts, the Gamos are manifested. The Gamos are not prone to chaos, extreme hate, and long-term conflict. The peaceful resolution of disputes between different interests and discussions is highly valued by them. As a result of the age-old experience, they are able to articulate with full confidence and authority about their grievances and tyranny on them as they present themselves in front of gathered people. It is possible for Gamo to present his thoughts with amazing tone, rhetoric, and coherence which is assumed developed from different sessions in Dubussha. It is primarily their side of the facts that will determine their confidence and power of persuasion in the Dubussha session. There is no loser in the Dubussha session; all sides are winners. CCE, from Dita Woreda, explained about the role of Dubussha in self-promotion and confidence that:

The Gamos are more likely than any other group to have a highly developed sense of engagement by making Dubussha have good qualities of self-expression, oratory characteristics, and speech compositions that are full of confidence and articulation. It is not only the Halqas of the Gamo people, but also the audience of Dubussha who learn how argumentation in discussion can be composed and presented with supporting parts of speech and tales. Making Dubussha while keeping turns using gestures (CHE, FN1, p. 3).

In the field note and an extensive interview with culture and communication experts and heritage workers in the Gamo zone, they all lauded for their ability to convince and influence their audience:

The Gamo elders are incredible at using words, diction, tone, argumentation, metaphors, and storytelling. With their gestures and cultural clothing, they always dominate the Dubussha session with charisma (CCE, FN1, p. 4).

#### 5.1.3 It is the essence of kindness

The Gamo people have developed the value of kindness over a generation, which has been passed on from generation to generation. Folktales and legends depict all creatures as being good for them. The hard work is highly valued and appreciated. The Dubussha of a certain district will take the lead whenever a man or woman faces a problem and provide all the solutions needed (CCE, FN1, p. 5).

In the history of the Gamo people, we had never faced such big economic or natural challenges that could threaten our integrity of kindness. Our Dubussha and Prier helped curtail bad things from being seen in our society. In our cultural system, we have a system of goodness for all creatures on the earth. This positive way of looking at nature is reflected in our settled life on earth. We (the Gamo people) have been blessed with productive land and wise human capital, which we have invested in kindness and fraternity.

### 5.2 Dubussha's role in environmental protection

In Dubussha sessions and practices, proverbs and sayings have more value in protecting and conserving the environment. Furthermore, for making Dubussha, the society planted special trees and preserved them for years. There are more than 650 Dubusshas in the Gamo Zone, which have trees and a conserved ecosystem where they discuss and deal with high-valued social issues. Furthermore, CCE and CHE have confirmed that the environmental protector who served Dubussha as environmental ambassador has the power to maintain forests and the environment in general. This cultural ambassador is called Demus/Demutha as a symbol of cutting trees is not good practice he prefers not cut off his hair until his death.

In the traditional Gamos structure, the Demus/ Demutha have divine power for protecting the environment. It is believed that no one can affect forests without his permission, and if it does, it will cause a curse (destruction). "Demutha" - according to informants, will perform a ritual or ritual of cleansing according to tradition, since such acts are considered crimes against the community. According to this traditional religious leader, social problems in the community will be resolved by appealing to the local peacemaker God (Xosso) to prevent future catastrophes (HHG, FN1 15).

### 5.3 Dubussha's roles to illiteracy and child labour abuse

Cultural values and norms are crucial components of science-based management for lasting and rooted change. With the help of research questions, this study was able to test the attitude and perceptions of participants in the FGD about illiteracy and child labour abuse using in-depth interviews. Among the key informants, most saw literacy as a waste of time, a violation of the traditional way of life, and a pandering to foreign ideas and customs. According to them, those young people who started working at an early age are likely to be dedicated workers and the most effective agents of change in society. The Gamos used to educate their children alternatively, but today there is a shift in understanding and practice, especially in urban areas of Gamo Zone, said Dita Woreda's culture and tourism expert:

Parents in our area pay more attention to teaching children’s hard work, cultural values, and social norms from the start. Therefore, we prefer taught them different tasks from the start as a society. It makes them more decent, helpful, and respectful if you keep them busy and show them their way of life at first in line with their culture it will be helpful. To make that effective we form dual system though the use of Dubussha. By doing so we have achieved results after our governmental structure start working with the cultural structure aggressively. By adopting the wills of Gamo elders, Halaqas and Hudugas which is the old structures with the new approach to child development and management through using Dubussha as a tool in Daramalo Worda the change we have observed was very impressive (CLE, FN1, p. 15).

The Gamos are a culturally dominated society; even today's culture has direct and indirect impact on any development intervention in the area. The imperative necessity of modernizing Dubussha and solving its social and economic problems cannot be underestimated. To this end, the interventions of different NGOs were not successful due to the lack of involvement and empowerment of Dubussha stakeholders.

Child labour abuse is rampant in the Gamo Zone in general in Chencha and Dita in particular. Although several projects are working on this issue, the practice was not stopped at the expected levels. All the instruments we have used so far have been heavily copied and customized from the experience of other countries which are far from our context. Therefore, we must work with cultural and communication experts to entertain cultural values and norms like Dubussha in order to influence culturally dominated societies (CLE, FN 1, P. 20).

An NGO expert working in this area has identified the interplay between child labor abuse and illiteracy in the Gamo zone as a cause of child labor abuse in the area.

Child labor abuse and illiteracy are two dominant challenges in the Gamo Zone. Especially child labor is the root cause of illiteracy and other deep-rooted problems in this area. We can reduce illiteracy and schooling rates if we work hard to stop child labor abuse. Several interventions have been launched in this regard, but all have been unsuccessful. Future social change projects should consider the use of Dubussha in such interventions (CCE, FN1, p. 21).

Most of the respondents (opinion followers) before the FGD were confident that their way of life and that of their forefathers were the most correct and perfect. In their view, their time was peaceful, trusting, and blessed. However, they have little understanding and little willingness to express their views on modern education and child abuse, which greatly affect their schooling. Therefore, as key informants claimed that:

We have a century-old skill of managing ourselves in our societal arrangement; among those growing children, it is one on which we place a high emphasis. Giving children more freedom is similar to leaving a tree alone after planting it. The plant will not bear fruit if proper care is not taken, just as we in Gamo society must teach our children to do what their fathers do (HHG, FN1, p. 18).

Unlike the FGD results, the understanding and intensity of children's imposition not to attend school varies by person, age, place, and level of cultural position. Individual differences are identified as a contributing factor in child illiteracy and child labor abuse in the Gamo Zone in general understanding due to access to education, proximity to zonal administration, family history about civilization and change, and openness to movement outside of their native society. Through his lived personal experience, one of the key informants explains why individual differences matter in Goza Kebele:

I grew up in the most culturally dominant area, which is the well-known "Goza Dubussha/ community discussion place" located 14 km from the district's main town Zada. Culture is the final decision maker in this area. The voices of the Halaqas and Hudagas are considered as God's voice. However, in terms of individual differences, our family has had some exposure to formal education and urban life. That motivates my father to send some of his children to school according to their age. This was not possible for all the children in Goza Kebele at the time, and it is still not easy today (CEE, FN1, p. 19).

Compared to banana-growing areas in the Gamo Zone, Chencha and Dita Woreda have a lower economic productivity and a lower standard of living. Several nongovernmental organizations (NGOs) and even the local government were ineffective in using the Haleqas and Hudugas, as well as the Gamos cultural communication platform-Dubussha, for developmental and societal change purposes. As a result, as the Dita zone culture and development expert expresses in the following quote, such intervention in the area is not as effective and productive as expected.

Child labor abuse and illiteracy have been identified as widespread in the Gamo Zone, particularly around Chencha and throughout Dita Woreda. Therefore, over the years, several non-governmental organizations (NGOs) have been deployed and working in this area. Despite the fact that their intervention is believed to be necessary in order to transform society in a more modern way of life. The society is also concerned about losing cultural values and norms in the name of modernism and civilization. To address these critical social questions and frustrations, using cultural opinion leaders and change makers is an unrivalled and productive method of intervention that both the government and NGOs in the area should adopt for any future development and social change interventions (CCE, FN, P. 20).

#### 5.3.1 Violating restrictions (Gome) and its results, reducing illiteracy and child

##### 5.3.1.1 Labour abuse

The Gamos have a deeply embedded conception, understanding, point of view, and philosophy with regard to natural, human, and social aspects. The divine rule and regulation recognized as Woga Wonta, which was first revealed on Dubussha the creation of humans, is exercised and arranged by all members of the society. As a result, every problem and change in the Gamos traditional governance system has traditional solutions (HHG, FN p. 17).

There is one expression in the Gamos golden saying that clearly depicts how the Gamos are open to change and the limit how they are ready for a new way of looking at accepts complementary views. Our ancestors said "Callous/Hand Mill grinds what you have given," which means that children, in particular, and Gamo society in general, are in the fens of the Woga Wonta. That is, by scaling up the Gamo system's productivity, attitude, and orientation, we can modernize and systematically transform our challenging parts of society on education and child labor abuse in the Gamo Zone at large (HHG, FN1, p. 18)

In any cultural society, it is obvious that a number of social fabrics exist to sustain the society's norms and values. It has lists and guidelines for the "Dos" and not the "Don'ts" as a society. Similarly, the Gamos have developed a strong cultural punishment and exclusion of wrongdoing tools known as the Violating restrictions/Gome in the Woga-Wonta system. Among other things, social inclusion is the most effective way to promote cultural change and improve communication. As a result, that inclusion stems from an understanding of its social fabrics, which typically include Dos and Don'ts in its societal guidelines. Social inclusion in the modern sense "It is a multifaceted process aimed at creating conditions that allow every member of society to participate fully and actively in all aspects of life, including civic, social, economic, and political activities, as well as participation in decision-making processes. Part II of the publication defines social inclusion as the process through which societies combat poverty and social exclusion [25].

Through systematic insertion and strategic communication, identifying and using culturally respected and acknowledged values and norms are very useful for participatory intervention and last long culture-based transformation. The intervention result of the present study showed that the Gamos Woga/violating restrictions rhetoric has the pervasive power to influence the society to reduce child labor abuse and children's illiteracy rates in a meaningful way. According to this finding according to Ref. [26], failure to integrate socially in any cultural barrier will result in social fragmentation; widening disparities and inequalities; and strains on individuals, families, communities, and institutions as a result of the rapid pace of social change, economic transformation, migration, and major population dislocations, particularly in areas of armed conflict. Even though we categorise societies as cultural or modern, it is unavoidable that cultural societies must pass through these six stages in order to be considered modern. Hunting and gathering societies, pastoral societies, horticultural societies, agrarian societies, industrial societies, and post-industrial societies have all existed in the world. The difference is how open the cultures of a given society are to change and how they cope with the dynamism of cultural change in the age of globalization [27].

As culture and communication experts assured the Gamo society has given much respect and due attention for restrictions on the Woga Wonta system. Fortunately, there are a number of stories and narratives that could align through the restriction of abusing children time and energy is cursed/Gome, as well as making them unwise and isolated it as an abnormal practice in the Gamo society at large. Through the consultation of Halaqas and Hudugas of Gamos, it is possible to bold those stories and narratives in the society day-to-day practice. By mainstreaming those narratives, it is possible to register positive and meaningful social change in Gamo society, the Dubussha cultural communication forum by using Gome as an instrument. CCE, FN-1, p. 18)

##### 5.3.1.2 The existence of dual power as an impediment to social change: reducing

###### 5.3.1.2.1 Illiteracy and child labor abuse

This study discovered that there is dual political power in most of the rural areas of the Gamo Zone. Cultural structures are more influential than government structures in rural areas such as Dita, Chencha, and Daramalo. As a result, if dual-power condensation is not handled in a more coordinated and wise manner, unfavourable outcomes may occur. To solve and reduce social challenges, cultural and governmental powers must work together. The government's commitment to using the cultural power in strategic change communication is found to be limited in this regard. Although it has the ability and potential to reduce illiteracy and child labour abuse in the zone, its role was not considered at the expected level.

Although the Gamo continue to have a say in the current national political system, informants say they have their own traditional organizational structure and political values that tie them to each door and that the Deres have a limited administrative structure based on their own good geographic content. This has resulted in 42 independent cultural government structures (Deres) run by these independent governments, a practice that has existed throughout history and is still present in some aspects of Gamo politics today. As a result, there are clearly two levels of government in the Gamo zone, particularly in rural areas. The Gamo Zone has two dual systems of governance: the government system and the traditional administrative system. Traditional administration is still more prevalent in some rural areas today. As stated by informants in Dita Woreda, there is a small tendency to collaborate with the government structure. However, in most rural areas, people prefer to take their cases to Dubussha and Halaqas rather than the government structure. The cultural expert adds to the difficulty created by the rural community's dual power struggle and the need to negotiate that conflict of interest.

Today, we have learned that it is critical to combine the traditional government system with the existing government structure in the zone area to achieve productive outcomes on a variety of development and social issues through consistent consultation between identified stakeholders through Dubussha and traditional communication platforms. Negotiating such power struggles and effectively leading in one direction is difficult unless this technique is initiated by using cultural systems as a gate for positive social change (CCE, FN1, p.14).

##### 5.3.1.3 Results of the intervention

When we analyzed the results of the intervention map, we began by interviewing all participants with FGD to assess their level of understanding about illiteracy and child labour abuse in their community. Except for the educated youths who participated in the FGD Halaqas and ordinary Gamos have relatively similar perception points on the interview stage, such as sending students to school on time and assigning those elongated tasks heavy duties.

This intervention prepared training for opinion leaders to see how modern thoughts can be generated through the traditional set-up in order to influence the masses. Training was difficult in terms of convincing Halaqas and/or Hudugas, but it was successful after a lengthy debate guided by the training manual. The FGD was then held without informing the other FGD participants that Halaqas and/or Hudugas had attended the training.

When Halaqas and/or Hudugas made their arguments about the importance of raising wise and modern children while respecting their cultural values, the students in education who participated in the FGD looked more satisfied and hopeful during the discussion. The Haleqas of Gamo were leading the discussion by substantiating their arguments with myths and proverbs; their influence was strong. Additionally, the ability of Gamo traditional leaders to lead, influence, and persuade was impressive. That quality is thought to have been developed through the Gamo's cultural communication forum Dubussha's age-old experience.

According to the findings of the focus group, the progress of the female participants was slow. So far, Gamo women have not been actively participating in the Dubussha session. They can only participate in this FGD if they can persuade the Haleqas and Man participants. Due to their limited access to Dubussha platforms, their participation and rhetorical organization of their thoughts were not as influential as Men's. The Gamos culture has strict rules that prohibit women from participating in Dubussha sessions throughout history.

As the finding further showed, the testing results which puts Woga system/Gome (cursing the violator of restrictions), have a magical multiplier effect on opinion followers in this intervention. When Haleqa's statements on the FGD were forwarded, most opinion followers were convinced that not sending students to school at the appropriate age and abusing them by assigning hard work is a cursed activity.

Several studies show how public opinion and behaviour change can be manipulated. According to Ref. [28], many people obtain their information and opinions from other people rather than from the media or other sources. These influential friends, family members, and acquaintances were dubbed "opinion leaders." The two-step flow hypothesis has been amended in a dozen ways to prefer influence over information, talk between equals over opinion leaders, multiple steps over two steps, etc. [29], but the consensus was that face-to-face relationships (between and among family, friends, neighbours, clan leaders) have such a strong influence on human behaviour [28].

Opinion leaders are change agents in a given society; any intervention that influences opinion leaders will most likely influence opinion followers. What we shall call opinion leadership, if we may call it leadership at all, is leadership at its simplest. It is casually exercised, sometimes unwittingly and unbeknownst, within the smallest grouping of friends, family members, and neighbours … It is the almost invisible, certainly inconspicuous, form of leadership at the person-to-person level of ordinary, intimate, informal, everyday contact, according to [28].

**Figure 3:**
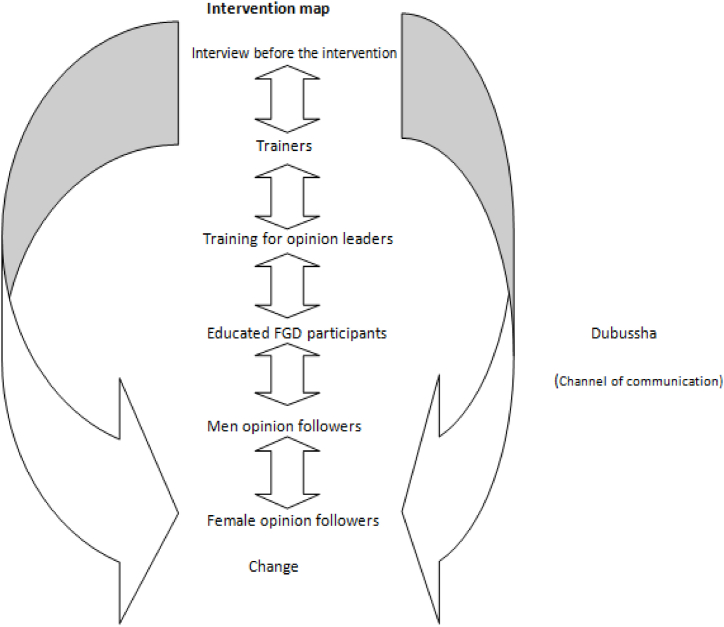
shows the summery result of the intervention^3^.

As explained in this study, the level and magnitude of change can differ when we introduce new ideas into the living system of a traditional society. According to Ref. [30], adopters of any new innovation or idea can be classified as innovators (2.5%), early adopters (13.5%), early majority (34%), late majority (34%), and laggards (16%). The influence of opinion leaders in this regard is critical and necessary (Fig. 3).

## 6 Conclusion and recommendation

### 6.1 Conclusion

Attempting to shed light on Gamo's forum for cultural communication, which is easily updated and can be used as a catalyst for social change in general and to get rid of the most pressing issues, like child labour exploitation and illiteracy in particular, was the primary goal of this study. Therefore, with an emphasis on reducing illiteracy and child labour abuse in Gamo Zone, Ethiopia, the study attempted to ethnographically investigate the essence, traditions of Dubussha use (trends), and communicative potential of Gamo's Dubussha as a cultural communication medium-as a traditional communication tool for communication of social change. The intervention, among other things, demonstrated that the "Woga Wonta/divine Gamos traditional government system" in general has deeply embedded cultural, economic, societal, and political practices and establishments in the people's psychosocial composition. As a result, this study looked into how this folk medium of communication can used to achieve societal change by emphasising the native's point of view.

By expressing opinions and sentiments through this platform, the Gamo culture ensures justice on all encompassing concerns in their area through daily Dubussha rituals. However, the main objective of this study was whether or not the use of Dubussha as a method to transmit a social change communication can reduce illiteracy and child labour abuse in the Gamo zone, Ethiopia. In a relative expression, a number of individuals were participated in testing of the research questions. They have been interviewed or invited discussants in FGDs held in people's natural surroundings, ranging from young and old participants to culturally experienced town residents working in various offices in the zone. When choosing study participants, socio-cultural and demographic aspects were also taken into account, as well as understanding the culture and custom of Gamo.

Finally, based on the study findings, the following conclusions were drawn. The Gamo people place a high value on their culture, and culturally based social actions in the Zone expected to be supported by that assumption. The Gamo people's "Woga Wonta/Divine System" is the dominating philosophy and ideology that underpins their entire traditional culture and government practices. These dominating guiding ideas have been passed down and practiced by various cultural positions such as Kawos, Haleqas, Hudugas, Bitanes, etc. Since the old time, the red line that all members of society should not cross has been known as Gome, and the assembly established by elders and traditional leaders has been known as Dulata. In Gamo culture, the cultural communication platform known as Dubussha is where all choices are made and implemented properly. The study also found that there is a variation in age, sex, cultural status, and ethnic identification when Dubussha is prepared. Even though there are no agenda restrictions in Dubussha assembly/Dileta, females and children do not participate in any Dubussha session.

### 6.2 Recommendation

In general, hardly every social topic has been adequately researched in a single study, and this study is neither exhaustive nor conclusive. The study did not address all of the issues that could have been addressed by using the Dubussha as a platform for cultural communication. Nonetheless, it made an effort to investigate the how, why, and what of Gamo's cultural communication forum, which can be easily updated and used as a catalyst for social change in general and the reduction of the most pressing issues, such as child labour exploitation and illiteracy in particular. The research only included a small population and sample in a relative expression. It is assumed that Ethiopia, a culturally diverse nation, is thought to use such straightforward and efficient communication techniques to promote long-lasting change. It is also crucial to keep in mind that understanding people's political, social, and cultural institutions is a prerequisite for implementing change that will have a lasting impact.

More crucially, any intervention in the Gamo Zone, particularly in its rural areas, should take into account local norms and values in order to encourage greater and faster success. Gender equality initiatives, health communication, malnutrition, criminal and conflict resolution, child labour abuse, and illiteracy reduction projects all require the use of cultural communication platforms, particularly Dubussha, as a venue for deep and long-lasting change. Furthermore, additional research and analysis are required to properly comprehend the role of women in the evolution of Gamo culture in general, and Dubussha in particular.

## Funding statement

This work was supported by Arba Minch University College of Social Sciences and Humanities (GOV/AMU/TH22/CSSH/DELL/01/2012).

## Data availability statement

The data will not be made available.

## CRediT authorship contribution statement

**Mekonen Teka Ayalew:** Writing – review & editing, Writing – original draft, Validation, Project administration, Methodology, Investigation, Funding acquisition, Formal analysis, Data curation, Conceptualization. **Tafesse Walea Wanke:** Validation, Project administration, Methodology, Investigation, Funding acquisition, Formal analysis, Data curation, Conceptualization.

## Declaration of competing interest

The authors declare that they have no known competing financial interests or personal relationships that could have appeared to influence the work reported in this paper.

## Footnotes

1 The picture above depicts a well-known cultural communication platform-Dubussha, also known as "Goza Dubussha/community discussion place," which is located 14 km from the district's main town Zada. The seats are arranged and established in such a way that they allow for appropriate communication and range of influence in the communication process. This also demonstrated that the Gamo society's age-old practice of deep-rooted culture and understanding for the potential of dialogue for any dispute is essential and irreplaceable.

2 The elders of the Gamo people gathered for discussion on the cultural communication platform- Dubussha. As it shown in the picture their arrangement in seat, use of material and selection of place and time is with meaning and rules in the Woga Wonta system.

3 Figure 1, a conceptual design of the intervention section of this study, was primarily created to present the summary and method of the intervention, FGD participants, communication channel, and outcomes in a precise manner.
